# The efficacy of regorafenib combined with PD-1/PD-L1 inhibitor in advanced or metastatic colorectal cancer: A single arm meta-analysis

**DOI:** 10.1097/MD.0000000000047284

**Published:** 2026-01-23

**Authors:** Fan Yang, Dandan Li, Baozhong Li, Ting Yang, Xiaoming Zhang, Zhiqiang Liu, Liang Zong

**Affiliations:** a Department of Central Laboratory, Changzhi People’s Hospital, The Affiliated Hospital of Shanxi Medical University, Changzhi, Shanxi Province, PR China; b Department of General Surgery, Anyang Tumor Hospital, Anyang, Henan Province, PR China; c Department of Gastrointestinal Surgery, Changzhi People’s Hospital, The Affiliated Hospital of Shanxi Medical University, Changzhi, Shanxi Province, PR China.

**Keywords:** advanced or metastatic colorectal cancer, meta analysis, PD-1/PD-L1 inhibitor, regorafenib

## Abstract

**Background:**

Regorafenib alone has shown limited efficacy in some cases of colorectal cancer (CRC), while the use of programmed cell death protein 1 (PD-1)/programmed cell death-ligand 1 (PD-L1) inhibitors is becoming increasingly common in cancer treatment. Previous studies have indicated significant benefits of combining regorafenib with PD-1/PD-L1 inhibitors in patients with advanced or metastatic CRC. This study aimed to evaluate the efficacy and outcomes of combining regorafenib with PD-1/PD-L1 inhibitors in patients with advanced or metastatic CRC.

**Methods:**

We systematically retrieved clinical trials from PubMed and Embase up to August 1, 2023. The quality of eligible clinical trials was assessed using the methodological index for non-randomized studies and the JBI critical appraisal checklist for case eeries. Efficacy metrics, such as objective response rate (ORR), disease control rate (DCR), median progression-free survival (mPFS), and median overall survival, were analyzed using STATA version 15.1, with results presented as 95% confidence intervals. Heterogeneity was assessed using the chi-square *Q* test and *I*² statistic, with *I*² > 50% indicating high heterogeneity, which was addressed using a random effects model.

**Results:**

Fourteen studies were included in this meta-analysis. The pooled ORR, DCR, and mPFS were 7%, 54%, and 2.99 months, respectively. Subgroup analysis revealed that for patients with liver metastasis (LM), the pooled ORR was 6%, DCR was 47%, and mPFS was 1.99 months. In contrast, for patients without LM, the pooled ORR was 21%, DCR was 61%, and mPFS was 3.48 months.

**Conclusion:**

These results indicate that combination therapy is more effective in patients without LM than in those with LM. This suggests that regorafenib plus PD-1/PD-L1 inhibitors may be a more suitable option for patients without liver metastases.

## 1. Introduction

Colorectal cancer (CRC) is one of the most prevalent malignant tumors worldwide. According to GLOBOCAN 2020, CRC ranks third in incidence and second in mortality among all cancers.^[1]^ Although early screening can substantially reduce CRC incidence, compliance rates remain suboptimal. Many patients with CRC present with metastases at initial diagnosis, leading to poor prognosis, reduced 5-year survival rates, and diminished quality of life.^[2]^ For resectable CRC, the treatment typically involves surgical resection combined with neoadjuvant chemotherapy. For unresectable cases, the Chinese Society of Clinical Oncology recommends palliative care including targeted therapy and immunotherapy.^[3]^

Recent advancements in immunotherapy have shown significant promise in cancer treatment by enhancing the ability of the immune system to target and destroy cancer cells. Programmed cell death protein 1 (PD-1)/programmed cell death-ligand 1 (PD-L1) inhibitors, as key immune checkpoint inhibitors, have demonstrated potent antitumor activity in various cancers. In clinical practice, PD-1/PD-L1 inhibitors, including nivolumab, avelumab, and pembrolizumab, are used for CRC patients with CRC.^[4]^ However, not all patients respond to PD-1/PD-L1 inhibitors, necessitating combination therapies to improve their efficacy.^[5]^

Regorafenib is a next-generation small-molecule multi-target tyrosine kinase inhibitor (TKI) that plays a critical role in antitumor angiogenesis and metastasis inhibition by targeting key kinases involved in cell proliferation, such as KIT, RAF, and RET. Several international clinical trials^[6-9]^ have evaluated the safety and efficacy of regorafenib in patients with advanced or metastatic CRC. But regorafenib is known as a drug with prominent treatment-related adverse events, including hand-foot skin reaction, diarrhea, and hypertension.^[10]^ Several studies have investigated the synergistic antitumor efficacy of regorafenib and PD-1/PD-L1 inhibitors in advanced or metastatic CRC was associated with multiple immune-related pathways in tumor microenvironment. Da-Liang Ou et al had demonstrated that regorafenib enhanced antitumor efficacy through reversed M2 polarization and adoptively transferred antigen-specific T cells.^[11]^ Dennis Doleschel et al investigated that the mechanism of egorafenib and PD-1/PD-L1 inhibitors was activation of cytotoxic T cells through activation of M1 macrophage and inhibition of Treg cell infiltration.^[12]^

But there also have limitations related to PD-1/PD-L1 inhibitor types, geographic variations, and sample sizes persist of regorafenib. This meta-analysis aimed to evaluate the efficacy of this combination therapy for advanced or metastatic CRC and provide valuable insights for clinicians in treatment decision-making.

## 2. Materials and methods

### 2.1. Search strategy

We conducted a comprehensive search for relevant studies in PubMed and Embase between 2000 and May 2023. The search used the following keywords and MeSH terms: “(((((Programmed Death Ligand 1 Inhibitors[Title/Abstract]) OR (PD-L1[Title/Abstract])) OR (avelumab[Title/Abstract])) OR ((((((((Programmed Cell Death Protein 1 Inhibitor[Title/Abstract]) OR (PD-1[Title/Abstract])) OR (nivolumab[Title/Abstract])) OR (toripalimab[Title/Abstract])) OR (pembrolizumab[Title/Abstract])) OR (sintilimab[Title/Abstract])) OR (camrelizumab[Title/Abstract])) OR (tislelizumab[Title/Abstract]))) AND (regorafenib[Title/Abstract])) AND ((Colorectal Cancer[[Title/Abstract]) OR (CRC[Title/Abstract])).” Additionally, we reviewed references from relevant literature and original articles to ensure that no eligible studies were overlooked.

### 2.2. Inclusion and exclusion criteria

#### 2.2.1. Inclusion criteria

Prospective clinical studies (including randomized controlled trials [RCTs] and single-arm studies) and retrospective studies. Articles investigating either PD-1/PD-L1 inhibitors or regorafenib, either as a single-agent therapy or in combination with other agents in patients with CRC.

Studies reporting efficacy metrics included overall response rate (ORR), disease control rate (DCR), median progression-free survival (mPFS), and median overall survival (mOS). The patients treated with more than 2 lines of standard chemotherapy regimens (such as fluorouracil, oxaliplatin, and irinotecan, with or without biological agents such as bevacizumab and cetuximab).

#### 2.2.2. Exclusion criteria

Article types: conference abstracts, letters, editorials, expert opinions, case reports, and reviews and studies lacking usable data, such as DCR and mPFS.

### 2.3. Data extraction

Two independent investigators reviewed and selected the studies for inclusion. Any disagreements were resolved by discussion with a third author. Data extracted from eligible studies included first author, study type, year of publication, sample size, patient enrollment dates, country, Eastern Cooperative Oncology Group performance status, median follow-up, and endpoints. The efficacy outcomes (ORR, DCR, mPFS, and mOS) were also extracted.

### 2.4. Quality assessment

Single-arm studies were evaluated using the methodological index for non-randomized studies. Retrospective reviews were conducted using the JBI critical appraisal checklist for case series.

### 2.5. Statistical analysis

Efficacy metrics (ORR, DCR, mPFS, and mOS) were analyzed using STATA version 15.1, with results presented as 95% confidence intervals (CIs). Heterogeneity was assessed using the chi-square *Q* test and *I*² statistic, where *I*² > 50% indicated significant heterogeneity, and a random effects model was employed. For studies reporting an ORR of 0, the Metaprop command was used. Publication bias was evaluated using the Begg’s and Egger’s tests.

## 3. Results

### 3.1. Study selection and characteristics

**Figure 1.**
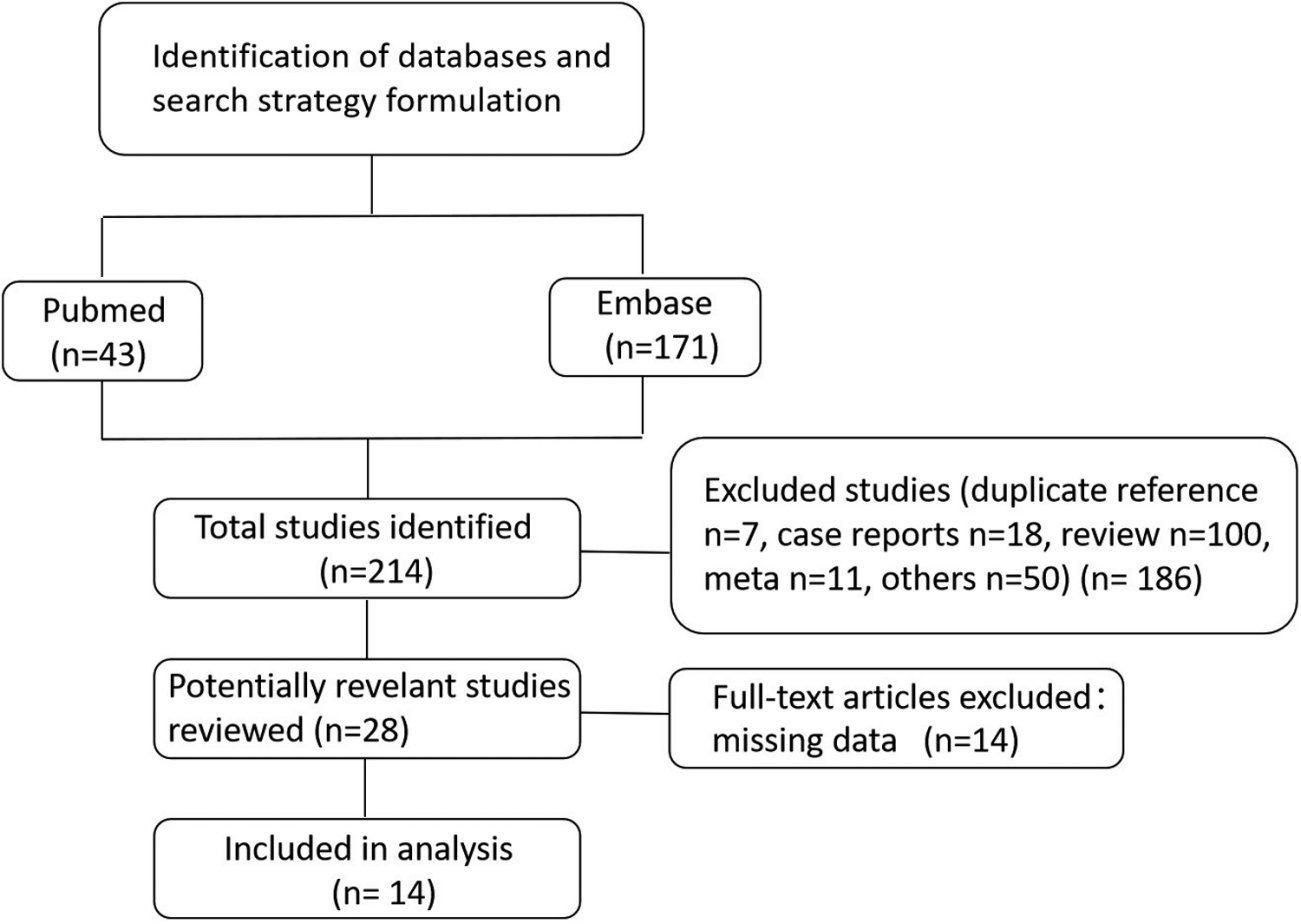
The flow diagram of this meta-analysis.

A total of 214 studies were initially identified through searches of PubMed and Embase from 2000 to May 2023. After excluding reviews, case reports, meta-analyses, and full-text articles, 14 studies met the inclusion criteria (Fig. 1). The included studies assessed PD-1/PD-L1 inhibitors such as nivolumab, toripalimab, pembrolizumab, sintilimab, camrelizumab, tislelizumab, and avelumab. The detailed characteristics of the studies are presented in Table 1.^[10,13-25]^

**Table 1:** Clinical characters of included studies.

Study, year	Country	Study type	ECOG	Median follow-up time (mo)	Previous lines of chemotherapy (%)	Therapeutic regimen	Sample size	Age (yr), median (range)	Male/female	Endpoint
Li et al, 2022	China	Retrospective study	0–1	7.8 (range 5.9–9.6)	Two lines 10 (43.5), 3 or more lines 13 (56.5)	Regorafenib plus anti-PD-1 antibodies	23	58.8 ± 10.6	18/5	OS, CR, PR, SD, and PD
Kim et al, 2022	United States	Open-label, single-arm phase I/Ib	0–1	NR	Two lines 30 (57.7), 3 or more lines 22 (42.3)	Regorafenib plus nivolumab	40	56 (31–79)	24/28	ORRs, OS, and PFS
Xu et al, 2022	China	Retrospective study	0–3	12	Two lines 14 (46.7), 3 or more lines 16 (53.3)	Regorafenib plus anti-PD-1 antibodies	30	57.5 (27–73)	14/16	ORR, CR, PR, SD, DCR, PFS, and OS
Chen et al, 2022	China	Retrospective study	0–2	16.2	Two lines 13 (54.2), 3 or more lines 11 (45.8)	Regorafenib plus anti-PD1 antibodies	24	68 (61–77)	9/15	OS, PFS, DCR, ORR, CR, PR, DCR, and SD
Li et al, 2020	China	Retrospective study	0–2	7.9 (95% CI 6.5–9.3)	Two lines 8 (34.8), 3 or more lines 15 (65.2)	Regorafenib plus anti-PD-1 antibodies	23	50 (33–73)	16/7	PFS, OS, ORR, DCR, CR, PR, SD, and PD
Li et al, 2022	China	Retrospective study	0–3	5.3 (range 0.5–22.5)	Two lines 58 (56.3), 3 or more lines 45 (43.7)	Regorafenib plus anti-PD-1 antibodies	55	56.0 (20.0–79.0)	56/47	OS, mPFS, treatment-related adverse events (TRAEs), CRs, PRs, and SD
Fukuoka et al, 2020	Japan	An open-label, and dose-expansion phase Ib	0–1	NR	Two lines 5 (20), 3 or more lines 20 (80)	Regorafenib plus nivolumab	25	55 (31–77)	18/7	ORR, DCR, PFS, and OS
Fakih et al, 2023	USA	A single-arm, open-label, multicentre phase 2	0–1	NR	Two lines 33 (47.1), 3 or more lines 37 (52.9)	Regorafenib plus nivolumab	70	57 (50–66)	41/29	ORR, AEs, SD, DCR, PFS, and OS
Wang et al, 2021	China	A phase Ib/II clinical trial	0–1	NR	Two lines 26 (66.7), 3 or more lines 13 (33.3)	Regorafenib plus toripalimab	39	53 (37–69)	20/19	ORR, DCR CR, PR, SD, PFS, and OS
He et al, 2023	China	Retrospective study		NR	Two lines 84 (100%)	Regorafenib plus anti-PD-1 antibodies	84	52 (42–59)	47/37	DCR, PFS, and adverse event
Cousin et al, 2021	France	A single-arm, open-label, phase II trial	0–1	7.2 (95% CI 6.4–8.1)	Two lines 20 (42.5), 3 or more lines 27 (57.4)	Regorafenib plus Avelumab	47	62 (26–83)	35/12	ORR, DCR CR, PR, SD, PFS, and OS
Yu et al, 2021	China	Retrospective study	0–1	NR	Two lines 16 (48.5), 3 or more lines 17 (51.5)	Regorafenib plus toripalimab	33	53.64 ± 10.34	15/18	ORR, DCR, CR, PR, SD, and PFS
Sun et al, 2021	China	Retrospective study	0–2	NR	Three lines 13 (56.5), 4 or more lines 10 (43.5)	Regorafenib plus anti-PD-1 antibodies	23	53.0 ± 12.02	14/9	ORR, DCR, CR, PR, SD, and PFS
Nie et al, 2022	China	Prospectively study	0–2	NR	Three lines 22 (52.4), 4 or more lines 20 (47.6)	Regorafenib plus sintilimab	42	59 (35–74)	18/23	ORR, DCR, CR, PR, SD, PFS, and OS

AE = adverse event, CI = confidence interval, DCR = disease control rate, ECOG = Eastern Cooperative Oncology Group, ORR = objective response rate, OS = overall survival, PD-1 = programmed cell death protein 1, PFS = progression-free survival, TRAE = treatment-related adverse event.

### 3.2. Quality assessment

Seven single-arm studies were evaluated using the methodological index for non-randomized studies and scored between 12 and 14 points, indicating high to moderate quality. Seven retrospective studies were assessed using the JBI critical appraisal checklist for case series (Table 2).

**Table 2:** Quality assessment of included studies.

MINORS index for included non-randomized studies																	
Study	I		II		III		IV		V		VI		VII		VIII		Total
Richard Kim	2		2		2		2		0		0		2		2		12
Shota Fukuoka	2		2		2		2		0		0		2		2		12
Marwan Fakih	2		2		2		2		0		2		2		2		14
Feng Wang	2		0		2		2		0		2		2		2		12
Sophie Cousin	2		2		2		2		0		2		2		0		12
Wei Yu	2		2		2		2		0		2		2		0		12
Caiyun Nie	2		2		2		2		0		2		2		0		12
JBI critical appraisal checklist for case series for included retrospective studies																	
Study	Q1	Q2		Q3		Q4		Q5	Q6	Q7		Q8		Q9		Q10	Overall appraisal
Lu Li	Y	Y		Y		Unclear		Y	Y	Y		Y		N		Y	Include
Yu-Jie Xu	Y	Y		Y		Y		Y	Y	Y		Y		N		Y	Include
Beibei Chen	Y	Y		Y		Y		Y	Y	Y		Y		N		Y	Include
Jisheng Li	Y	Y		Y		Y		Y	Y	Y		Unclear		N		Y	Include
Rong-Rong Li	Y	Y		Y		Y		Y	Y	Y		Unclear		N		Y	Include
Liying Sun	Y	Y		Y		Y		Y	Y	Y		Unclear		N		N	Include
Wenzhuo He	Y	Y		Y		Unclear		Y	Y	Y		Y		N		Y	Include

I. A clearly stated aim; II. Inclusion of consecutive patients; III. Prospective collection of data; IV. Endpoints appropriate to the study aim; V. Unbiased assessment of the study endpoint; VI. Follow-up period appropriate for the aim of the study: VII. Loss to follow-up <5%; VIII. Prospective calculation of the study size. The items are scored as 0 (not reported), 1 (reported but inadequate), or 2 (reported and adequate).

Q1. Were there clear criteria for inclusion in this case series? Q2. Was the condition measured in a standard and reliable way for all participants included in the case series? Q3. Were valid methods used to identify the condition of all participants included in the case series? Q4. The case series included consecutive inclusion of participants. Q5. Did the case series have complete inclusion of participants? Q6. Was there clear reporting of the demographics of the participants in the study? Q7. Was there clear reporting of the clinical information of the participants? Q8. Were the outcomes or follow-up results of the cases reported clearly? Q9. Was there clear reporting of the presenting site(s)/clinic(s) demographic information? Q10. Was the statistical analysis appropriate?

MINORS = methodological index for non-randomized studies.

### 3.3. Therapeutic efficacy assessments

#### 3.3.1. Tumor response

**Figure 2.**
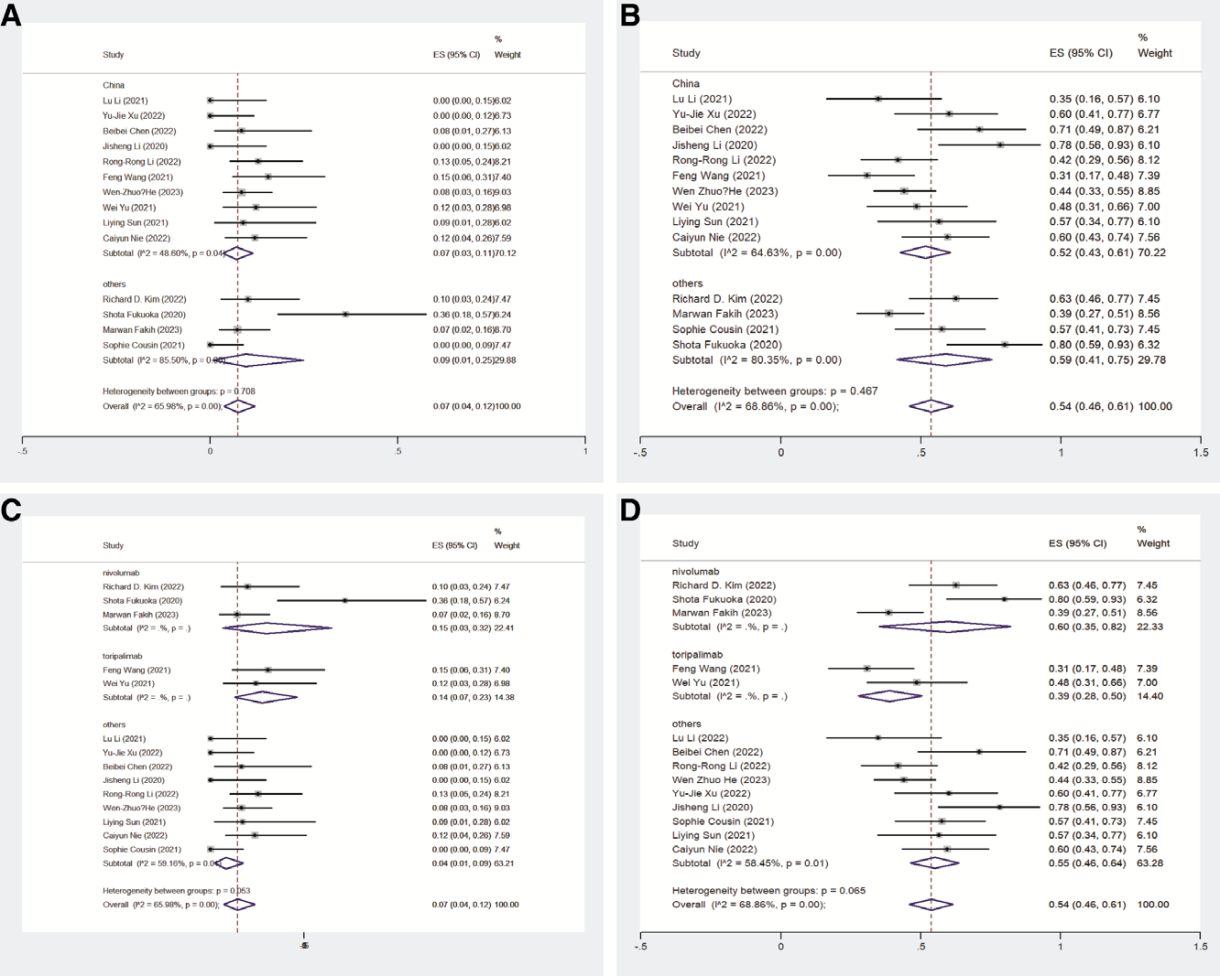
The tumor response. (A, B) Pooled ORR and pooled DCR of China and other countries; (C, D) pooled ORR and pooled DCR of different PD-1 antibodies. DCR = disease control rate, ORR = overall response rate, PD-1 = programmed cell death protein 1.

The ORR from the 14 studies was 7% (95% CI: 4–12%) with significant heterogeneity (*I*² = 65.98%, *P* = .000). Among these, studies conducted in China reported a pooled ORR of 7% (95% CI: 3–11%) with moderate heterogeneity (*I*² = 48.6%, *P* = .04), whereas studies conducted abroad reported an ORR of 9% (95% CI: 1–25%) with high heterogeneity (*I*² = 85.5%, *P* = .00). DCR data from the 14 studies yielded a pooled DCR of 54% (95% CI: 46–61%) with significant heterogeneity (*I*² = 68.86%, *P* = .00). Subgroup analysis revealed a pooled DCR of 52% (95% CI: 43–61%) in studies from China and 59% (95% CI: 41–75%) in studies conducted abroad (Fig. 2).

When analyzing specific PD-1/PD-L1 inhibitors, the pooled ORR for nivolumab, toripalimab, and other inhibitors were 15% (95% CI: 3–32%), 14% (95% CI: 7–23%), and 4% (95% CI: 1–9%), respectively, with significant heterogeneity (*I*² = 59.16%, *P* = .01). The corresponding DCRs were 60% (95% CI: 35–82%), 39% (95% CI: 28–50%), and 55% (95% CI: 46–64%), with significant heterogeneity (*I*² = 58.45%, *P* = .01; Fig. 2).

#### 3.3.2. Survival

**Figure 3.**
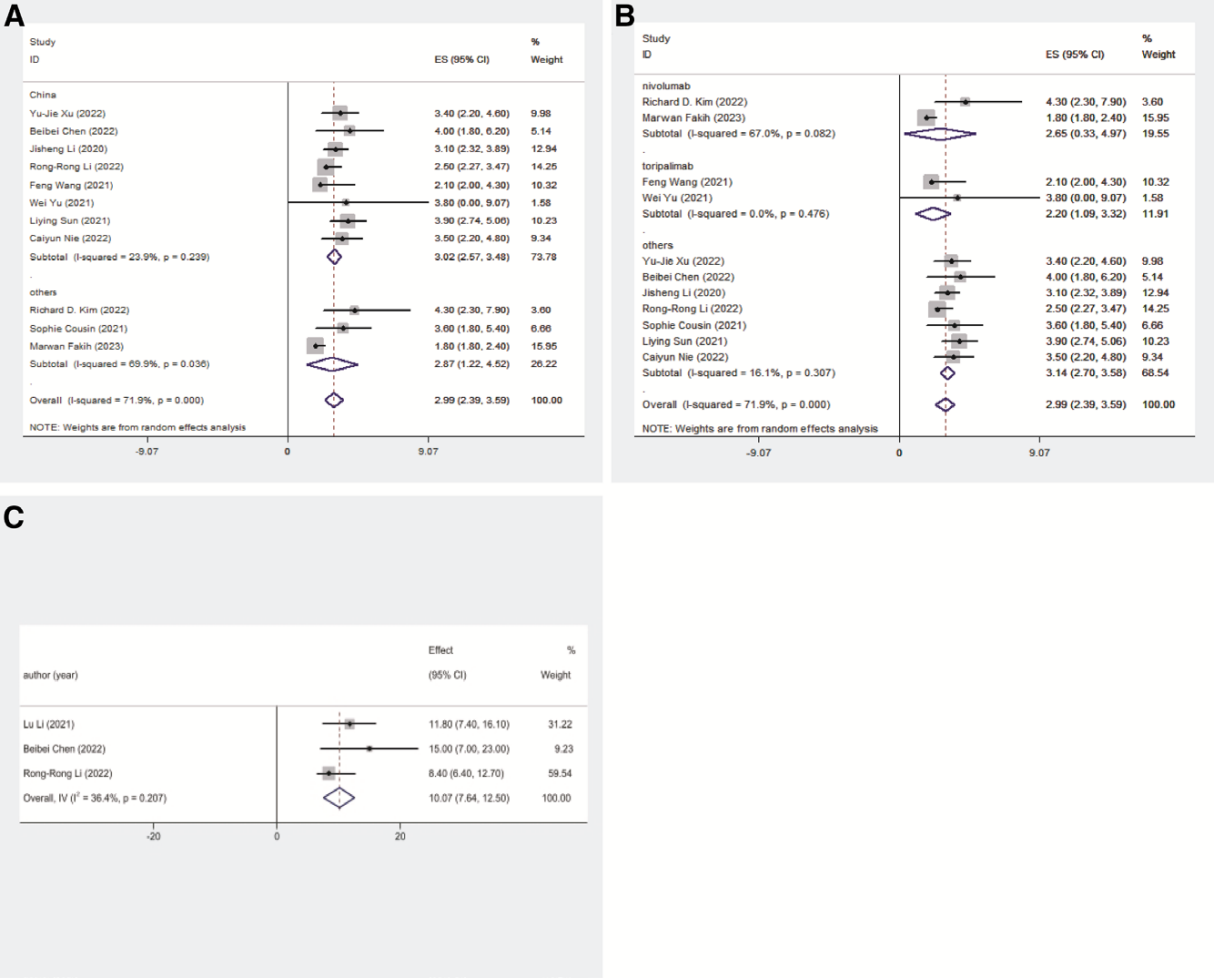
The survival results. (A) Pooled mPFS of China and other country; (B) pooled mPFS of different PD-1 antibodies; and (C) pooled mOS of China. mPFS = median progression-free survival, mOS = median overall survival, PD-1 = programmed cell death protein 1.

Eleven studies reported mPFS with a pooled mPFS of 2.99 months (95% CI: 2.39–3.59) and significant heterogeneity (*I*² = 71.9%, *P* = .000). Subgroup analysis showed that studies conducted in China had a pooled mPFS of 3.02 months (95% CI: 2.57–3.48) without significant heterogeneity (*I*² = 23.9%, *P* = .239), whereas studies abroad reported an mPFS of 2.87 months (95% CI: 1.22–4.52) with significant heterogeneity (*I*² = 69.9%, *P* = .036). Analysis by PD-1/PD-L1 inhibitor type revealed varied mPFS: toripalimab had a pooled mPFS of 2.20 months (95% CI: 1.09–3.32) without significant heterogeneity (*I*² = 0.0%, *P* = .476); other inhibitors (pembrolizumab, sintilimab, camrelizumab, tislelizumab, nivolumab and avelumab) had a mPFS of 2.65 months (95% CI: 0.33–4.97) with significant heterogeneity (*I*² = 67.0%, *P* = .082); and other inhibitors (pembrolizumab, sintilimab, camrelizumab, tislelizumab, avelumab) had a pooled mPFS of 3.14 months (95% CI: 2.70–3.58) without significant heterogeneity (*I*² = 16.1%, *P* = .307; Fig. 3).

The mOS was reported in 3 studies, with a pooled mOS of 10.07 months (95% CI: 7.64–12.50) for Chinese patients (Fig. 3).

#### 3.3.3. Subgroup analysis

**Figure 4.**
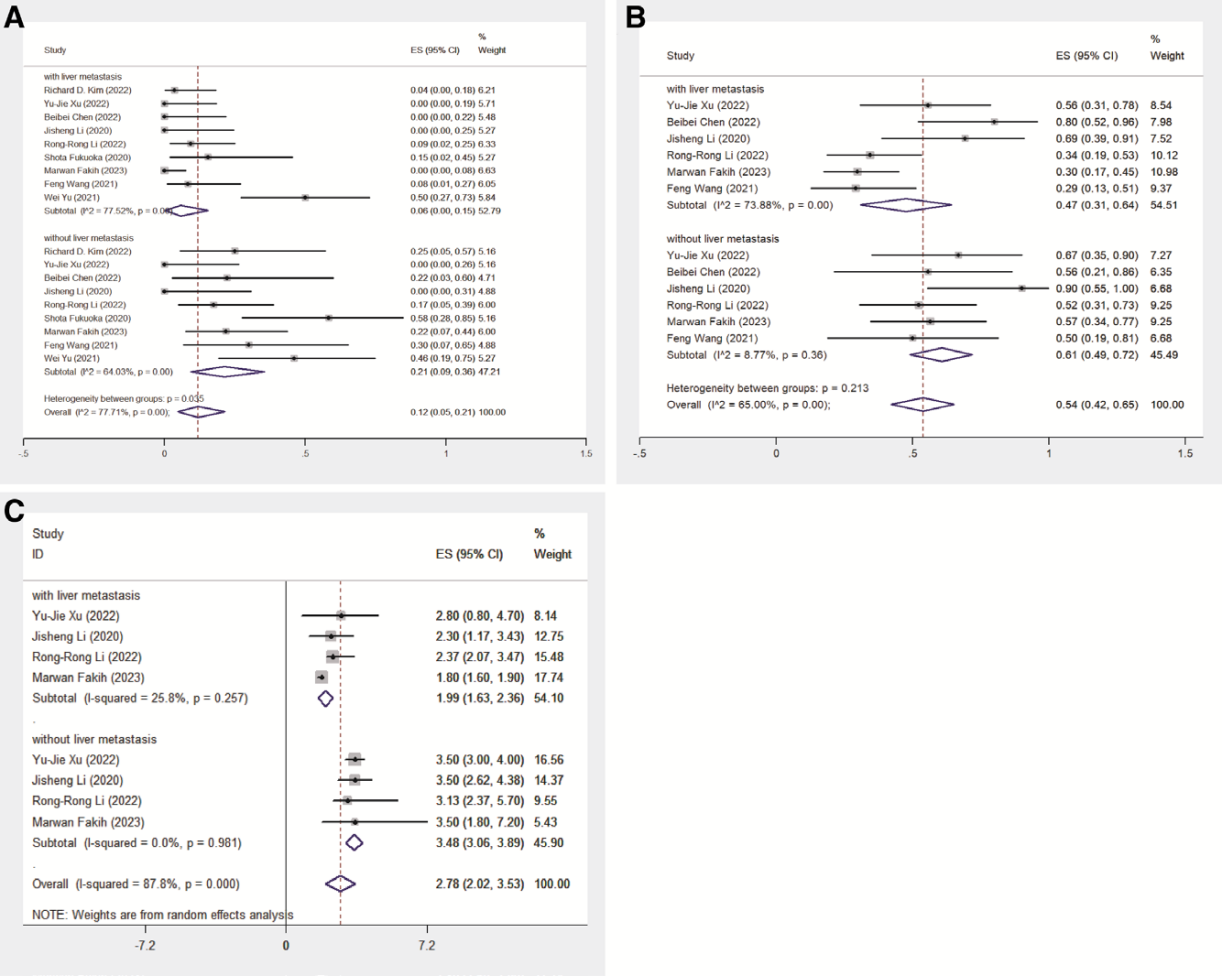
The tumor response and survival of subgroup. (A) The pooled ORR of with LM subgroup and without LM subgroup; (B) the pooled DCR of with LM subgroup and without LM subgroup; and (C) the pooled mPFS of with LM subgroup and without LM subgroup. DCR = disease control rate, mPFS = median progression-free survival, ORR = overall response rate.

Nine studies investigated patients with liver metastasis (LM) versus those without LM treated with regorafenib and PD-1/PD-L1 inhibitors. The pooled ORR was 12% (95% CI: 5–21%), with significant heterogeneity (*I*² = 77.71%, *P* = .00). The ORR for patients with LM was 6% (95% CI: 0–15%) with significant heterogeneity (*I*² = 77.52%, *P* = .00), whereas for patients without LM, it was 21% (95% CI: 9–36%) with moderate heterogeneity (*I*² = 64.03%, *P* = .00; Fig. 4).

The DCR for subgroups with and without LM was reported in 6 studies. The pooled DCR was 54% (95% CI: 42–65%), with significant heterogeneity (*I*² = 65.00%, *P* = .00). Subgroup analysis revealed that patients without LM had a higher pooled DCR of 61% (95% CI: 49–72%) with no significant heterogeneity (*I*² = 8.77%, *P* = .36) than those with LM, who had a DCR of 47% (95% CI: 31–64%) with significant heterogeneity (*I*² = 73.88%, *P* = .00; Fig. 4).

Four studies reported the mPFS in subgroups with and without LM. The pooled mPFS was 2.78 months (95% CI: 2.02–3.53) with significant heterogeneity (*I*² = 87.8%, *P* = .000). Patients without LM had a significantly longer pooled mPFS of 3.48 months (95% CI: 3.06–3.89) compared to 1.99 months (95% CI: 1.63–2.36) for those with LM. Neither group showed significant heterogeneity (*I*² = 0.0%, *P* = .981 for without LM; *I*² = 25.8%, *P* = .257 for LM; Fig. 4).

## 4. Discussion

The recent studies showed that the CRC incidence and mortality in Europe, Oceania, and northern America was decreased. However, data from the National Cancer Center of China in 2022 showed the incidence and mortality of CRC increased in China. Therefore, efficient treatment strategy was crucial for CRC patients.^[26–29]^ Various treatment regimens have been explored to address these issues, including primary tumor resection combined with chemotherapy,^[30]^ oral multi-targeted TKIs with or without PD-1/PD-L1 inhibitors,^[15,31]^ and other therapeutic agents.^[32]^ Recent advances in immunotherapy, particularly with immune checkpoint inhibitors, have shown potential in improving the treatment outcomes for several cancers.^[33]^ Regorafenib, a TKI, is used in CRC, hepatocellular carcinoma, osteosarcoma, and gastrointestinal stromal tumors, among others.^[34–36]^ However, its efficacy is not universal and some patients do not benefit from this treatment. The combination of regorafenib with PD-1/PD-L1 inhibitors represents a novel approach for advanced or metastatic CRC, but most studies are single-arm, phase I or II trials, or retrospective reviews with small sample sizes. This study extracted efficacy data, including ORR, DCR, and mPFS; however, mOS data were not available. Also, the adverse events was collected from the 14 articles in Supplementary Table https://links.lww.com/MD/R185, such as fatigue, diarrhea, hand-foot skin reaction, hypertension, liver dysfunction.

The systemic immune desert was presented in liver metastases with preclinical models. This study divided the patients into subgroups based on the presence of LM. In patients with LM, the ORR was 6% compared to 21% in those without LM. The DCR was 47% in the LM subgroup and 61% in the non-LM subgroup, indicating better efficacy in patients without LM. The mPFS was 1.99 months in patients with LM and 3.48 months in those without LM, reflecting a longer survival time and better prognosis in patients without LM. Some studies showed the potential mechanism for the tumor of LM. Yu et al reported that patients with liver metastases derive limited benefit from immunotherapy through siphon activated CD8+ T cells from systemic circulation.^[37]^ Liu et al demonstrated that the infiltration of Kupffer cells played role in antitumor responses by nibbling cancer cells and skewing toward proinflammatory macrophages.^[38]^ Wu et al reported that MRC1 + CCL18 + M2-like macrophages was activated in a single-cell and spatial atlas of colorectal LM, whereas neoadjuvant chemotherapy decreased the activation.^[39]^

This study had several limitations. Firstly, the data exhibited considerable heterogeneity. Additionally, publication bias is present (Supplementary Figure https://links.lww.com/MD/R185) due to factors such as small sample sizes, the inclusion of unpublished data, and reliance on single-arm, phase I, or II studies. Future updates to this meta-analysis may benefit from the inclusion of RCTs, as additional data become available.

## 5. Conclusion

The use of PD-1/PD-L1 inhibitors or regorafenib as standalone treatments does not demonstrate high efficacy in advanced or metastatic CRC. Therefore, combining PD-1/PD-L1 inhibitors with regorafenib requires further investigation. This study found that the ORR, DCR, and mPFS were superior in patients without LM than in those with LM. Future research should include larger sample sizes and RCTs to provide more robust evidence for the efficacy of these treatments.

## Author contributions

**Conceptualization:** Liang Zong.

**Data curation:** Fan Yang, Dandan Li.

**Formal analysis:** Baozhong Li.

**Funding acquisition:** Liang Zong.

**Writing – original draft:** Fan Yang, Dandan Li.

**Writing – review & editing:** Baozhong Li, Ting Yang, Xiaoming Zhang, Zhiqiang Liu, Liang Zong.

## Supplementary Material


