# Supplementary material for: The efficacy of regorafenib combined with PD-1/PD-L1 inhibitor in advanced or metastatic colorectal cancer: A single arm meta-analysis
Source: Medicine (Baltimore). 2026 Jan 23;105(4):e47284. doi: 10.1097/MD.0000000000047284 (PMC12851704; doi:10.1097/MD.0000000000047284)
Supplement: Supplementary file 1 [file medi-105-e47284-s001.docx]

Supplementary Figure. Publication bias.


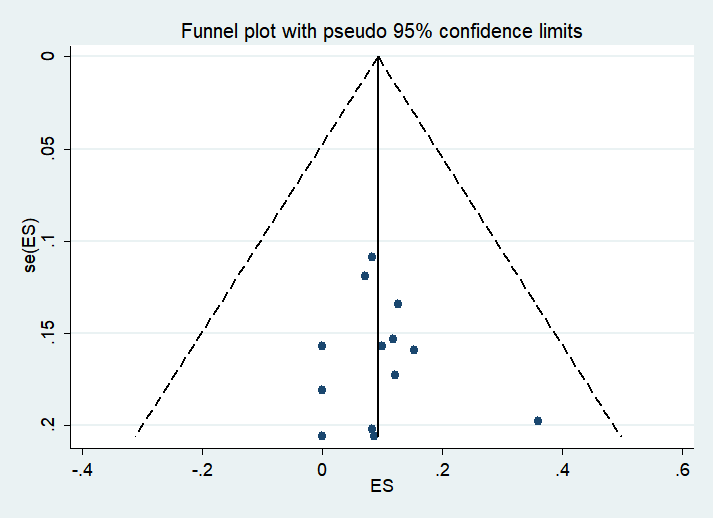


Supplementary Table Side events of regorafenib combined with PD-1/PD-L1 inhibitor

| Study, year | Any grade (≥10%) | ≥grade3 (≥3%) |
| --- | --- | --- |
| Li et al, 2022 | - | - |
| Kim et al, 2022 | Fatigue (51), Palmar-plantar erythrodysesthesia Syndrome (41.2%), Diarrhoea (39.2%), Rash (33.3%), ALT increase (29.4%), Hypophosphatemia (29.4%), Hypertension(15.2%) | Hypertension (15.7%), Rash (9.8%), Lymphopenia (7.8%), Anaemia (5.9%), Palmar-plantarerythrodysesthesiasyndrome (3.9%) |
| Xu et al, 2022 | hand-foot syndrome (33.3%), hypertension (23.3%), malaise (20.0%), gastrointestinal reaction (16.7%), transaminase elevation (13.3%), diarrhea (10.0%) | hand-foot syndrome (6.7%), abnormal capillary proliferation (3.3%), transaminase elevation (3.3%) |
| Chen et al, 2022 | fatigue, diarrhea., hand-foot skin reaction (12.5%), hypertension (4.2%), and proteinuria (4.2%) | hand-foot skin reaction, hypertension, and oral mucositis |
| Li et al, 2020 | palmar-plantar erythrodysesthesia(39.1%), hypertension (26.1%), fatigue (43.4%), liver dysfunction (21.7%), and decreased appetite (17.4%). | Palmar-plantar erythrodysesthesia (8.7%), rash (4.3%), liver dysfunction (4.3%) |
| Li et al, 2022 | AST increase (27.2), ALT increase (25.2), TBIL increase (22.3), Hemoglobin decreased (14.6), Palmar-plantar erythrodysesthesia (13.6), Hypothyroidism (12.6) | aspartate aminotransferase (AST) increase (3.8%) |
| Fukuoka et al, 2020 | Palmar-plantar erythrodysesthesia (70%), Hypertension (48%), Rash (42%), Fatigue (40%), Fever (40%), Proteinuria (32%), Liver dysfunction (18%) | Proteinuria (12%), Palmar-plantar erythrodysesthesia (10%), Rash (12%), Liver dysfunction (6%), Hypertension (4%) |
| Fakih et al, 2023 | fatigue (16%), decreased appetite (9%) | fatigue (4%), Maculopapular rash (3%) |
| Wang et al, 2021 | Hand-foot syndrome (51.3%), rash (30.8%), fever (20.5%), hoarseness (17.9%), diarrhea (17.9%), hypertension (15.4%), impaired liver function(15.4%), chest distress (15.4%), myalgia (12.8%), headache (12.8%), thrombocytopenia (10.3%), and fatigue (10.3%). | Hand-foot syndrome (10.3%) and impairedliver function (10.3%) |
| He et al, 2023 | hand-foot syndrome (33.3%), rash (13.1%), and hepatotoxicity (11.9%) | None |
| Cousin et al, 2021 | Fatigue (62%), anorexia (57%), palmar-plantar erythrodysesthesia (45%), Oral mucositis (40%), Dysphonia (38%), diarrhea (36%), AST and/or ALT increased (30%), Blood bilirubin increase (28%), Hypertension (19%), Myalgia (17%) | Palmo-plantar erythrodysesthesia (30%), Hypertension (23%), fatigue (6%), Oral mucositis (4%), Diarrhea (13%), AST and/or ALT increased 14 (13%), Blood bilirubin increase 13 (4%) |
| Yu et al, 2021 | hand-foot syndrome (33.33%), liver dysfunction (27.27%), hypothyroidism (24.24%), fever (24.24%),  fatigue (21.21%), leukopenia (15.15%), hypertension(12.12%) | Hand-foot syndrome (3.03%), Myocarditis (3.03%), Liver dysfunction (3.03%) |
| Sun et al, 2021 | liver dysfunction (52.2%), palmar–plantar erythrodysesthesia (43.5%), hypertension (39.1%), RCCEP (39.1%), proteinuria (30.4%), and fatigue (30.4%) | palmar–plantar erythrodysesthesia (4.3%), rash (4.3%), liver dysfunction (4.3%), colonic perforation (4.3%), and myocardial enzyme elevation(4.3%) |
| Nie et al, 2022 | fatigue (31.9%), decreased appetite (30.6%), secondaryhypertension (23.6%), hypothyroidism (20.8%), oral mucositis (19.4%), diarrhea (13.9%), hand foot syndrome (11.1%) | secondary hypertension (5.6%) |
